# The Role of Crosstalk between AR3 and E2F1 in Drug Resistance in Prostate Cancer Cells

**DOI:** 10.3390/cells9051094

**Published:** 2020-04-28

**Authors:** Jin Xu, Xi Yang, Dhanraj Deshmukh, Hegang Chen, Shengyun Fang, Yun Qiu

**Affiliations:** 1 Department of Pharmacology, University of Maryland School of Medicine, Baltimore, MD 21201, USA; jinxu@umaryland.edu (J.X.); Xyang@som.umaryland.edu (X.Y.); dhanrajdeshmukh@umaryland.edu (D.D.); 2 Veterans Affairs Medical Center, Baltimore, MD 21201, USA; hegang.chen@som.umaryland.edu; 3 Department of Epidemiology and Public Health, University of Maryland School of Medicine, Baltimore, MD 21201, USA; 4 Center for Biomedical Engineering and Technology, Department of Physiology, Marlene and Stewart Greenebaum Comprehensive Cancer Center, University of Maryland School of Medicine, Baltimore, MD 21201, USA; sfang@som.umaryland.edu

**Keywords:** prostate cancer, combination therapy, drug resistance, androgen receptor splicing variants, E2F1, auranofin

## Abstract

Background: Drug resistance is one of the most prevalent causes of death in advanced prostate cancer patients. Combination therapies that target cancer cells via different mechanisms to overcome resistance have gained increased attention in recent years. However, the optimal drug combinations and the underlying mechanisms are yet to be fully explored. Aim and methods: The aim of this study is to investigate drug combinations that inhibit the growth of drug-resistant cells and determine the underlying mechanisms of their actions. In addition, we also established cell lines that are resistant to combination treatments and tested new compounds to overcome the phenomenon of double drug-resistance. Results: Our results show that the combination of enzalutamide (ENZ) and docetaxel (DTX) effectively inhibit the growth of prostate cancer cells that are resistant to either drug alone. The downregulation of transcription factor E2F1 plays a crucial role in cellular inhibition in response to the combined therapy. Notably, we found that the androgen receptor (AR) variant AR3 (a.k.a. AR-V7), but not AR full length (AR-FL), positively regulates E2F1 expression in these cells. E2F1 in turn regulates AR3 and forms a positive regulatory feedforward loop. We also established double drug-resistant cell lines that are resistant to ENZ+DTX combination therapy and found that the expression of both AR3 and E2F1 was restored in these cells. Furthermore, we identified that auranofin, an FDA-approved drug for the treatment of rheumatoid arthritis, overcame drug resistance and inhibited the growth of drug-resistant prostate cancer cells both in vitro and in vivo. Conclusion and significance: This proof-of-principle study demonstrates that targeting the E2F1/AR3 feedforward loop via a combination therapy or a multi-targeting drug could circumvent castration resistance in prostate cancer.

## 1. Introduction

Prostate cancer is one of the most frequently diagnosed cancers in men. According to the American Cancer Society, 191,930 new cases and 33,330 deaths have been estimated for prostate cancer in United States for 2020. Hormonal therapy is the standard treatment for advanced prostate cancer patients. Unfortunately, the tumors eventually acquire androgen-independence and progress to castration-resistant prostate cancer (CRPC). Docetaxel (DTX) is the first line of treatment for androgen-independent metastatic CRPC. However, a large portion of CRPC patients do not respond to DTX. The new-generation androgen receptor (AR)-targeting agent, enzalutamide (ENZ), significantly increased the overall survival of CRPC patients, either with or without previous chemotherapy [1,2]. However, a similar primary and acquired resistance was observed in ENZ-treated CRPC patients. It is important to note that the majority of patients rapidly developed resistance in less than two years after treatment. Thus, drug resistance—especially resistance to multiple drugs—has become the most significant barrier to overcome, both in the laboratory and in the clinic.

DTX is a taxane compound, and exerts its cytotoxic function via the stabilization of guanosine diphosphate (GDP)-bound tubulin in microtubules. It is widely accepted that the stabilization of microtubules can cause mitotic spindle dis-regulation, thereby leading to the death of rapidly-dividing cancer cells. Microtubules also play important roles in intracellular protein trafficking. The microtubule motor protein dynein is critical for the nuclear translocation of AR, while the taxane-induced microtubule stabilization inhibits the nuclear accumulation and transcriptional activity of AR [3]. The present study indicates that taxane compounds can exert a prostate cancer-specific anti-tumor function by inhibiting AR signaling. However, AR seems to interact with dynein via a C-terminal ligand binding domain according to several recent studies [4,5]. The constitutively-activated AR splicing variant, AR3 (a.k.a. AR-V7), lacks the C- terminal ligand binding domain of AR, and thus the nuclear localization and transcriptional activity of AR3 is not affected by microtubule-targeting agents [4]. This finding, in conjunction with our data, demonstrating that AR3 expression is increased in CRPC, indicates that AR3 could play an important role in the resistance to DTX. ENZ is a nonsteroidal antiandrogenic compound that targets the C-terminal ligand binding domain of AR. Thus, AR3 cannot be targeted by ENZ. Likewise, the detection of AR3 in circulating tumor cells is associated with ENZ resistance in CRPC patients [6]. Overexpression of AR3 in 22Rv1 prostate cancer cells decreased the ENZ-induced inhibition of cell growth [7]. These data suggest that AR3 may play an important role in resistance to multiple monotherapies.

In this study, we found that the expression of E2F1 was substantially altered in response to a combined DTX+ENZ treatment in cells that were resistant to either drug alone. It has been reported that E2F expression was downregulated by DTX in HN12 and HN30 head and neck squamous cell lines [8], and our previous microarray results showed that the expression of E2F family transcripts was elevated in a DTX-resistant prostate cancer cell line [9]. Importantly, E2F1 is also a critical downstream regulator in response to ENZ. Furthermore, ENZ has been show to suppress the expression of the E2F1 target genes DHFR and TK1, and genes regulated by E2F1 were significantly enriched in an ENZ-resistant prostate cancer cell line [10]. E2F1 is a dual role regulator in cancer progression, where increases in E2F1 can either lead to apoptosis or promote tumor growth and invasion in cancers [11]. In response to DNA damage, E2F1 can either induce apoptosis or stimulate DNA repair. In addition, E2F1 is closely associated with AR function, both as an oncogene and a tumor suppressor. Elevated levels of E2F1 are associated with AR hyperactivity under conditions of androgen blockage [12]. E2F1 directly interacts with AR and regulates the expression of downstream genes in an androgen-dependent manner [13]. Moreover, androgen signaling promotes E2F1-induced cell cycle progression by disrupting the interaction of E2F1 with its co-suppressor PHB [14]. In contrast, Mallik et al. [15] demonstrated that the interaction of E2F1 with AR was ligand independent, and that adding androgen decreased the E2F1-AR interaction. The function of the AR-E2F1 axis is highly dependent on the status of Rb. Gao et. al. [16] reported that AR directly repressed genes related to DNA replication and DNA repair through the recruitment of the Rb protein in response to androgen treatment, which is mediated by the N-terminal region of AR. As constitutively-activated AR3 shares the same N-terminal with AR, it is very possible that AR3 and the E2F1-Rb complex have both physical and functional associations. Thus, it is important to investigate the role of AR3 and E2F1 in drug-resistant prostate cancers.

Therefore, the aim of the present study was to examine the effect of a combined DTX+ENZ therapy on prostate cancer cells that are resistant to either drug alone. Furthermore, we sought to determine the functional relationship between E2F1 and AR3, and the role of the E2F1/AR3 feedforward loop in drug resistance. This research provides new insights into the mechanisms underlying drug resistance in pre-clinical models.

## 2. Materials and Methods

### 2.1. Cell Culture and Drug Treatment

R1/DTX was developed as previously described [17]. R1-ADR (CWR-R1 derived cells resistant to DTX and androgen-deprivation therapy (ADT)) was developed from R1/DTX and cultured in charcoal-stripped serum (CSS) containing Roswell Park Memorial Institute (RPMI) 1640 medium for three months in the presence of 1nM DTX, and were maintained under the same conditions. AI, a LNCaP derivative cell line, was cultured long-term in CSS medium, and was a gift from Dr. Wenliang Li from the University of Texas Health Science Center at Houston. LN-ADR (LNCaP derived cells resistant to DTX and ADT) was developed by culturing the AI cells in the CSS-containing medium and treating with DTX. The concentration for selection was determined by treating the cells with 0.1, 1, 2, 5, and 10 nM DTX for three days. Based on the cell survival rate (about 50%) after treatment for three days, 1nM DTX was used. In three months, the cells could grow in the presence of 1nM DTX at a similar doubling time to AI cells, and they were then maintained under these conditions. ENZ-resistant R1-ENZR (CWR-R1 derived cells resistant to ENZ) cells were developed by culturing the CWR-R1 cells in CSS-containing medium in the presence of 40 μM ENZ. In three months, the cells could grow in the presence of 40 μM ENZ at a similar doubling time to CWR-R1 cells, and they were then maintained under these conditions. R1-DDR (CWR-R1 derived cells resistant to DTX and ENZ) and LN-DDR (LNCaP derived cells resistant to DTX and ENZ) double drug-resistant cell lines were developed from R1-ADR and LN-ADR, respectively. Double drug-resistant cell lines were cultured in CSS containing RPMI 1640 medium in the presence of 1nM DTX and 10 μM (R1-DDR) or 20 μM (LN-DDR) ENZ. In three months, the cells could grow in the presence of 1nM DTX and 10 μM (R1-DDR) or 20 μM (LN-DDR) at a similar doubling time to R1-ADR or LN-ADR cells, respectively, and they were then maintained under these conditions. Drug resistant prostate cancer cell lines used in the present study were cultured in CSS-containing medium and were subcultured regularly when confluent unless otherwise mentioned. All other cell lines were initially purchased from American Tissue Culture Collections (ATCC, Manassas, VA, USA)). HEK-293T cells were cultured in Dulbecco’s modification of Eagle’s medium (DMEM) supplemented with 10% FBS. Transfection experiments were carried out using FuGENE HD (Roche Applied Science, Penzberg, Germany) according to the manufacturer’s instructions. DTX was kindly provided by Sanofi Aventis. Enzalutamide (S1250) was purchased from Selleckchem (Houston, TX, USA) and dissolved in DMSO (stock concentration: 50 mM). Rucaparib (S1098) was purchased from Selleckchem (Houston, TX, USA) and dissolved in DMSO (stock concentration: 10 mM). Cabazitaxel (S3022) was purchased from Selleckchem (Houston, TX, USA) and dissolved in DMSO (stock concentration: 10 mM). Auranofin (BML-EI206-0100) was purchased from Enzo Life Sciences (Farmingdale, NY, USA) and dissolved in DMSO (stock concentration: 200 mg/mL).

### 2.2. Constructs, Antibodies, and shRNA

The plasmids used in the present study are as follows: the E2F1 expression vector pCMVHA E2F1 was a gift from Kristian Helin (Addgene, Cambridge, MA, USA, plasmid # 24225) [18], the E2F1 DNA binding mutant vector pCMV E2F1 E132 was a gift from Kristian Helin (Addgene, Cambridge, MA, USA, plasmid # 24224) [19], the E2F1 promoter luciferase vector pGL2-AN was a gift from William Kaelin (Addgene, Cambridge, MA, USA, plasmid # 20950) [20], and the AR expression vector and ARR2-Luc vector were generated as previously described [21,22].

The antibodies used in this study include: Rabbit polyclonal anti-AR3 [22], Anti-AR3(Clone RM7; ReMab, San Francisco, CA, USA), Anti-AR-FL (sc-815; Santa Cruz, Dallas, TX, USA), Anti-AR (sc-816; Santa Cruz, Dallas, TX, USA), Anti-α-tubulin (clone DM1A; Sigma, St Louis, MO, USA), Anti-E2F1 (clones KH20 and KH95; Millipore, Burlington, MA, USA), Anti-E2F1 (A300-766A; Sigma, St Louis, MO, USA), Anti-Rb (9309S; CST, Danvers, MA, USA), Anti-PARP1(sc-8007; Santa Cruz, Dallas, TX, USA), Anti-Caspase3 (9661S; CST, Danvers, MA, USA), Anti-GAPDH (sc-365062; Santa Cruz, Dallas, TX, USA), and Anti-Ki67 (9449; CST, Danvers, MA, USA).

The shRNAs used in this study include:

shAR-FL was purchased from Sigma.

shAR3:

TAGGCTAATGAGGTTT ATTTCTCAAGAG AAAATAAACCTCATTAGCCTTTTTTTTTC

shE2F1-1:

CCGGCTACTCAGCCTGGAGCAAGAACTCGAGTTCTTGCTCCAGGCTGAGTAGTTTTTG

shE2F1-2:

CCGGCGCTATGAGACCTCACTGAATCTCGAGATTCAGTGAGGTCTCATAGCGTTTTTG

### 2.3. Cell growth, Spheroid, and Apoptosis Assay

Cell growth was measured using cell counting kit-8 (CCK8) assay (Vita Scientific, Beltsville, MD, USA). Cells were seeded in 96-well plates (Thermo Fisher Scientific, Waltham, MA, USA) at a density of 2000 cells/well. Drugs were added the following day (D1), and then refreshed every two days for 3–6 days as indicated in each experiment. Then, 10 µL of the CCK8 solution was added to each well and the cells were cultured for 1.5 h at 37 °C. Absorbance was measured using a plate reader at 450nm wavelength. Five wells were used for each experimental condition. Spheroid formation was assessed using Ultra-low attachment multiple well plate (CLS7007-24EA; Corning/Costar, Corning, NY, USA). Spheroid size was evaluated using the free software ImageJ. Eight thousand cells were suspended in a final volume of 100 μL/well and cultured for four days. Then, half of the old medium was replaced by fresh medium containing the appropriate amounts of agents every two days and cultured for 14 days. Finally, a TUNEL apoptosis assay was performed using the DeadEnd™ Fluorometric TUNEL System (Promega, Madison, WI, USA) according to the manufacturer’s instructions.

### 2.4. RNA-Seq

Cells were lysed in QIAzol lysis reagent (Qiagen, Hilden, Germany). Total RNA was extracted using a RNeasy mini kit (Qiagen, Hilden, Germany) according to the manufacturer’s instructions. RNA-seq was performed by Arraystar, Inc (Rockville, MD, USA). Briefly, total RNA from each sample was quantified using a NanoDrop ND-1000. Subsequently, 1–2 μg of total RNA was used to prepare the sequencing library according to the following steps: 1. Total RNA was enriched by oligo (dT) magnetic beads (rRNA removed); and 2. RNA-seq library was prepared using the KAPA Stranded RNA-Seq Library Prep Kit (Illumina, San Diego, CA, USA), which incorporates dUTP into the second cDNA strand and renders the RNA-seq library strand-specific. The completed libraries were qualified using an Agilent 2100 Bioanalyzer and quantified using the absolute quantification qPCR method. To sequence the libraries, the barcoded libraries were mixed, denatured to single stranded DNA in NaOH, captured on Illumina flow cell, amplified in situ, and subsequently sequenced for 150 cycles at both ends using the Illumina HiSeq 4000. RNA-seq data was deposited in NCBI’s Sequence Read Archive (BioProject Accession: PRJNA623560). Analyses were performed using BRB-ArrayTools, which was developed by Dr. Richard Simon and the BRB-ArrayTools Development Team. Changes in gene expression were examined by two-tailed two-sample t-test and the Benjamini-Hochberg procedure [23] was used to derive false discovery rates (FDR). Meanwhile, the fold changes of genes were calculated. We also acknowledge our use of the gene set enrichment analysis (GSEA) software and Molecular Signature Database (MSigDB) [24,25].

### 2.5. Quantitative Real-Time PCR (qRT-PCR) and Chromatin Immunoprecipitation (ChIP)

Total RNA was extracted using a RNeasy Mini kit (Qiagen, Hilden, Germany) and reverse transcribed using Transcriptor Reverse Transcriptase (Roche, Penzberg, Germany). Quantitative real-time PCR was performed as previously described [26].

The qRT-PCR primer sequences used in this study include:

AR-FL:

F: ctactccggaccttacggggacatgcg R:gggctgacattcatagccttcaatgtgtgac

AR3:F:CTACTCCGGACCTTACGGGGACATGCG R: TGCCAACCCGGAATTTTTCTCCC

E2F1: F: AGCTGGACCACCTGATGAAT R: GTCCTGACACGTCACGTAGG

Actin: F: GCTCGTCGTCGACAACGGCTC R: CAAACATGATCTGGGTCATCT

Immunoprecipitation was performed as previously described [21]. 1 × 10^8^ R1-ADR cells were used for each ChIP. The ChIP primer sequences used in this study include:

ChIP-E2F1-P: F: GTTGGGGGCTACAGGTTGAG R: AAGTCCCGGCCACTTTTACG

ChIP-FKBP5: F: GCATGGTTTAGGGGTTCTTGC R: AACACCCTGTTCTGAATGTGGC

ChIP-AR-siteA: F: GACTCGCAAACTGTTGCATT R: TACAGCACTGGAGCGGCTA

ChIP-AR-siteB: F: CCTAGCAGGGCAGATCTTGT R: TCCCCTTCTCTTGCTCAGAA

ChIP-AR-siteC: F: GGTAGGAAGTGGCTGAATTCTGGATGA R: CCCTGCCCATGCACCTGCTC

### 2.6. Luciferase Reporter Assay

Luciferase assays were carried out as previously described [22]. Briefly, the HEK293T cells were transfected with AR-FL, AR3, E2F1, or the E2F1 E132 mutant expression plasmids and E2F1-Luc or ARR2-Luc in various combinations as indicated. The cells were then cultured in DMEM medium. Twenty four hours after transfection, the cells were treated with ENZ or Dihydrotestosterone (DHT) for 24 h, and dual-luciferase assays were performed according to the manufacturer’s instructions (Promega, Madison, WI, USA). The results are presented as the relative changes in luciferase activity.

### 2.7. Co-Immunoprecipitation and Immunofluorescence

For HEK-293T exogenous IP, the cells were transfected with the appropriate plasmids and allowed to grow for 48 h in 100 mm dishes. For R1-ADR endogenous IP, the cells were plated in P150 dishes for three days, 1nM DHT was added, and the cells were incubated for 4 h before being collected as indicated. 3 × 10^8^ cells were used for each IP. Immunoprecipitation was performed as previously described [27]. Immunofluorescence was performed as previously described [27].

### 2.8. Xenograft Model

The animal studies were performed according to protocol ##0917012 (approved on 09/20/2019) reviewed and approved by the Institutional Animal Care and Use Committee (IACUC) at the University of Maryland School of Medicine, Baltimore, MD, USA. The R1-DDR xenograft was carried out as previously described [22]. Animals were treated with solvent or DTX (5 mg/kg once per week) + ENZ (10 mg/kg per day, 5 days per week) or auranofin (5 mg/kg per day, 5 days per week), n = 5 per group. Tumor sizes and body weight were measured every Monday and Thursday. Tumor volumes were calculated according to the following formula: 0.52 × r_1_^2^ × r_2_ (r_1_ < r_2_). Results of tumor volumes were expressed as means ± SEM. The effect of treatment was analyzed by one-way ANOVA. Post-hoc analysis was performed using Tukey’s test. Relative body weight was analyzed by repeated measures ANOVA with group as between subject factor and time as within subject factor. Immunohistochemical staining of mouse xenograft tumors was carried out as previously described [21].

## 3. Results

### 3.1. DTX-ENZ Combined Treatment Inhibited Cell Growth and Induced Apoptosis in Prostate Cancer Cells Resistant to DTX or ENZ

**Figure 1:**
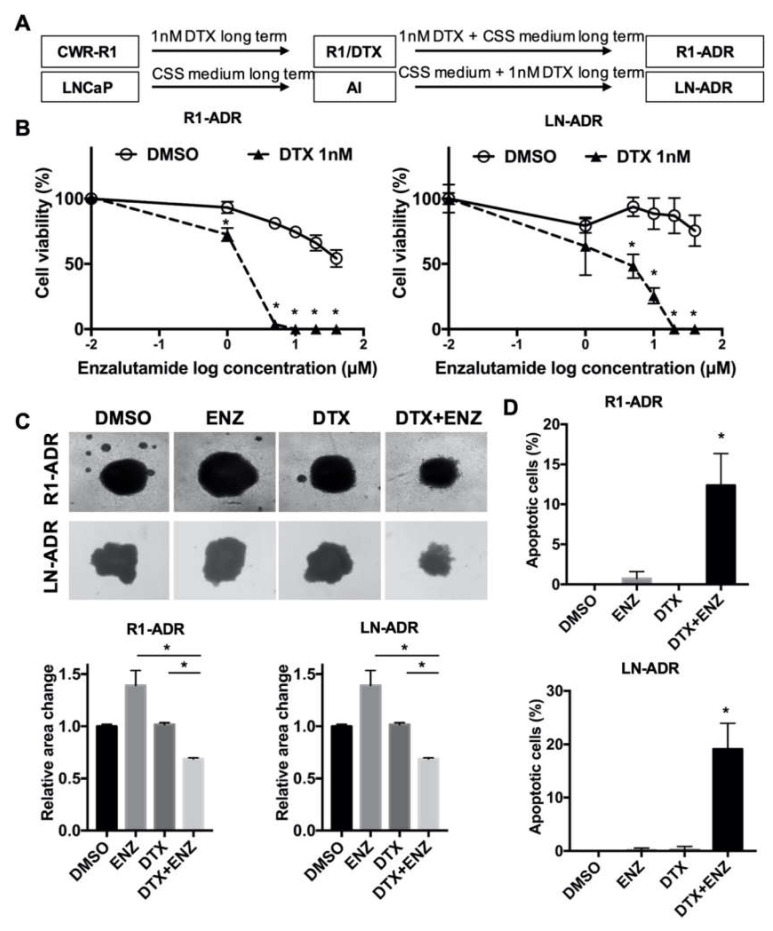
DTX-ENZ combined treatment inhibited growth of drug resistant R1-ADR and LN-ADR cells. (**A**) Schematic diagram of the establishment of drug resistant cell lines. (**B**) Cell growth was determined by CCK-8 assays. R1-ADR (left panel) and LN-ADR (right panel) cells were treated with different doses of ENZ for 6 days with or without 1nM DTX. (**C**) Morphology (upper panel) and quantification (lower panel) of tumor spheroids after 14 days of treatment with 40 μM ENZ and/or 2nM DTX (R1-ADR) or 4nM DTX (LN-ADR). (**D**) TUNEL assays were performed to detect apoptosis under the indicated treatments in R1-ADR (upper panel) and LN-ADR (lower panel) cells after 48 h treatment. Cells were treated with 1nM DTX and 10 μM ENZ (R1-ADR) or 20 μM ENZ (LN-ADR). Error bars, S.D. * *p* < 0.05.

To develop potential combined therapies for ADT- and DTX-resistant prostate cancer cells, we first generated the ADT- and DTX- resistant prostate cancer cell models, R1-ADR and LN-ADR, which were derived from CWR-R1 and LNCaP parental cells, respectively (Figure 1A). It is widely accepted that switching to ENZ significantly prolonged the survival of patients after treatment with DTX [2]. Thus, we examined the effect of ENZ on DTX-resistant R1-ADR and LN-ADR cells. Both cell lines exhibited very limited responses to ENZ treatment (Figure 1B). However, in the presence of 1nM DTX, the efficacy of ENZ was enhanced at least 20 fold in both cell lines, which exhibited much lower IC50 values (R1-ADR: IC50 **≈** 1.4 μM, LN-ADR: IC50 **≈** 2.6 μM) in comparison to ENZ treatment alone (R1-ADR: IC50 **≈** 53.4 μM, LN-ADR: IC50 **>** 50 μM). We also showed that the combined DTX+ENZ therapy exerted a better inhibitory effect than DTX alone in ENZ-resistant R1-ENZR cells (Figure S1A). These results indicate that the synergistic effects of these two drugs are independent of the order of prior monotherapies. In addition, we also tested various other drug combinations, including the taxane compound cabazitaxel and the PARP inhibitor rucaparib (Figure S1B,C), CK1/2 inhibitors (D-4476/CX-4945), GSK3 inhibitors (CHIR-99021), and an AKT inhibitor (perifosine) (unpublished data). However, those drug combinations did not display any synergistic effects, suggesting that the DTX+ENZ combination may be a unique treatment to overcome DTX or ENZ resistance.

Next, we evaluated the inhibitory effect in a tumor spheroid model. Similar to the effect in 2D cell culture, the combination of DTX and ENZ inhibited 3D-spheroid growth to a greater extent than did DTX or ENZ alone in R1-ADR and LN-ADR cells (Figure 1C). To assess whether the inhibitory effect of the DTX+ENZ combined therapy was the result of apoptosis, we performed a TUNEL assay with different drug treatments. The combination of DTX and ENZ exhibited a significantly higher number of TUNEL-positive cells in comparison to either drug alone (Figure 1D, Figure S1D). To further confirm this finding, apoptosis markers for the levels of cleaved PARP and cleaved caspase-3 were measured by western blot, and were consistently increased in the combined therapy group in both cell lines (Figure S1E). Taken together, these results indicate that the combination of DTX and ENZ inhibited the growth and induced apoptosis of drug-resistant prostate cancer cells.

### 3.2. Differential Gene Expression in Response to DTX-ENZ Combined Treatment of Prostate Cancer Cells

**Figure 2:**
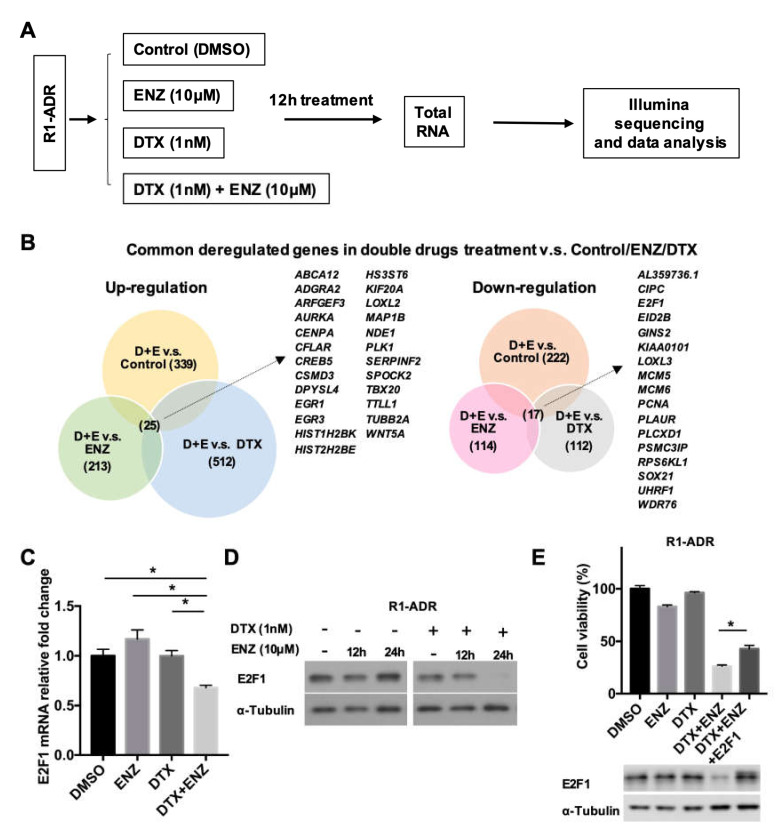
Differential gene expression in response to DTX-ENZ combined treatment. (**A**) Flowchart of RNA sample preparation and RNA-seq. (**B**) Analysis of common deregulated genes in double drugs v.s. Control or ENZ or DTX group. (*p* < 0.05, FDR < 0.25, fold change >1.4) (**C**) Expression of E2F1 in R1-ADR cells 12 h after drug treatment were determined by qRT-PCR. Error bars, S.D. * *p* < 0.05. (**D**) Protein expression of E2F1 in R1-ADR cells 12 and 24 h after drug treatment were determined by western blot. (**E**) Re-expressed E2F1 partially rescued DTX+ENZ induced growth inhibition in R1-ADR cells. Cell growth was determined by CCK-8 assays 72 h after re-introduced exogenous E2F1 in the presence of DTX and ENZ. Error bars, S.D. * *p* < 0.05.

To investigate the molecular mechanism and cellular pathways responsible for the inhibition of growth observed in response to the combined treatment, we performed RNA-seq to identify differentially-expressed genes in R1-ADR cells after 12 h of drug treatments (Figure 2A). We were specifically interested in changes in gene expression in the combined treatment, as neither DTX nor ENZ alone inhibited growth. First, we performed a GSEA to compare DTX+ENZ with the other groups (control, DTX alone, or ENZ alone). In agreement with the TUNEL, which showed an increase in apoptosis in the combined treatment group, we found that apoptosis-related hallmarks were enriched in the DTX+ENZ combination group using GSEA (Figure S2A). Next, we compared patterns of gene expression between DTX+ENZ and the control, DTX alone, or ENZ alone. An analysis of RNA-seq data identified 25 genes that were commonly upregulated and 17 genes that were commonly downregulated in DTX+ENZ treatment groups in comparison to other groups (Figure 2B), many of which were found to be related to cell cycle processes, DNA replication, and DNA repair. To further narrow down the possible changes in gene expression responsible for the inhibition of growth as a result of the combined treatment, we analyzed altered genes in KEGG pathways in cancer. Our analysis demonstrated that several pathways—including CREB5, WNT5A, and E2F1—were all altered in response to the DTX+ENZ combined treatment in comparison to either drug alone (Figure S2B). Of these genes, E2F1 is an important transcriptional mediator in cancer progression and has both oncogenic and tumor-suppressive properties.

To assess whether E2F1 is critical in the progression of prostate cancer, we analyzed publicly available clinical databases by cancer type using cBioPortal. We found a relatively high rate of genomic amplification or mutation of E2F1 in several types of cancers, including prostate cancer (Figure S2C). A further analysis of E2F1 in prostate cancer showed that E2F1 was highly amplified in selective prostate cancer studies (Figure S2D). A Kaplan-Meier survival analysis of the TCGA prostate adenocarcinoma dataset using the UCSC Xena platform indicated a trend of survival disadvantage for patients with high E2F1 expression (top 25%) in comparison to those with low E2F1 expression (bottom 25%) (Figure S2E). Thus, as an increase in E2F1 expression plays an important role in advanced prostate cancer, the growth inhibition from the DTX+ENZ combined treatment could be caused by the suppression of E2F1 activity. The downregulation of E2F1 expression in response to the combined treatment was validated by qRT-PCR (Figure 2C). In agreement with the qRT-PCR results, E2F1 protein reduction was detected by western blot of prolonged combined treatment in R1-ADR and LN-ADR cells (Figure 2D, Figure S2F). Furthermore, growth inhibition as a result of the combined treatment can be partially rescued by exogenously expressed E2F1 in R1-ADR cells (Figure 2E). Taken together, these data strongly suggest that E2F1 is an important pro-survival mediator in drug resistant cells.

### 3.3. AR Variant and AR-FL Differentially Regulated E2F1 Expression in Drug-Resistant Cells

**Figure 3:**
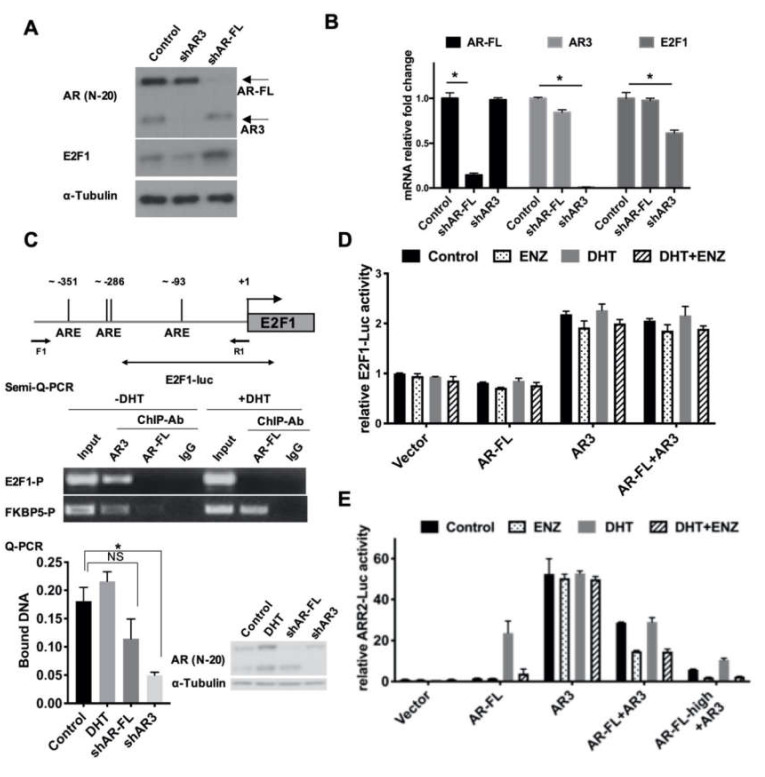
AR3 and AR-FL differentially regulated E2F1 expression. (**A**,**B**) R1-ADR cells were infected with the lentivirus encoding the control shRNA, AR3 shRNA (shAR3) or AR-FL shRNA (shAR-FL) for 48 h. Protein levels of E2F1, AR-FL, and AR3 were determined by western blot (**A**) and mRNA levels were determined by qRT-PCR (**B**), Error bars, S.D. * *p* < 0.05. (**C**) Binding of AR3 or AR-FL to the putative ARE sites of human E2F1 gene regulatory region was analyzed by ChIP assays. Error bars, S.D. * *p* < 0.05. (**D**) The E2F1-promoter driven luciferase reporter (E2F1-LUC) activity was measured in HEK293T cells treated with 0.1nM DHT or/and 10 μM ENZ. (**E**) ARR2-promoter luciferase assay was performed in HEK293T cells treated with 0.1nM DHT or/and 10 μM ENZ.

Because AR-FL is a known target of ENZ, we wondered whether AR or its splice variant, AR3, played a role in the regulation of E2F1 expression. To test this hypothesis, we knocked down AR-FL or AR3 expression in R1-ADR cells using target-specific shRNAs. The level of E2F1 protein was decreased only in AR3 knock-down cells (Figure 3A). To determine whether AR3 regulated E2F1 on the transcriptional level, we examined E2F1 mRNA levels via qRT-PCR. In agreement with the western blot results, we found that the levels of E2F1 mRNA were decreased in AR3 knock-down cells (Figure 3B). To further examine whether AR3 directly regulates E2F1 expression by binding to E2F1 regulatory regions in chromatin, we analyzed the E2F1 promoter sequence and identified several putative androgen response elements (AREs). We then performed a ChIP assay using anti-AR3 and anti-AR-FL antibodies and found that AR3, but not AR-FL, binds to the E2F1 promoter. The specificity of AR3 binding was confirmed by knocking down AR3, but not AR-FL, which effectively diminished the recruitment of AR3 to the E2F1 promoter (Figure 3C). The FKBP5 regulatory region was used as a positive control (Figure S3A). In addition, the treatment of R1-ADR cells with 1, 10, or 100 nM of DHT had little effect on E2F1 protein expression (Figure S3B), suggesting that AR-FL and androgen signaling play a very limited role in regulating E2F1 expression in these cells. Next, we examined the activity of AR-FL and AR3 using an E2F1 promoter-driven luciferase reporter (E2F1-LUC). As shown in Figure 3D, only AR3, but not AR-FL, enhanced E2F1 reporter activity, which was not affected by the addition of AR-FL or treatment with DHT or ENZ (Figure 3D, Figure S3C). In the meantime, AR3 and AR-FL could enhance the canonical androgen pathway target ARR2-LUC reporter activity (Figure 3E, Figure S3D). Although AR3 itself was sufficient to induce ARR2-LUC reporter activity, in the presence of AR-FL, ENZ effectively lowered the AR3-induced ARR2-LUC activity, which is in agreement with previous studies [28]. Taken together, these data suggest that AR3 regulates E2F1 expression independent of AR-FL in R1-ADR cells, and its dependency on AR-FL in regulating gene expression may be context dependent.

### 3.4. Differential Regulation of E2F1 by AR3 and AR-FL is Mediated by the Recruitment of Co-Factors

**Figure 4:**
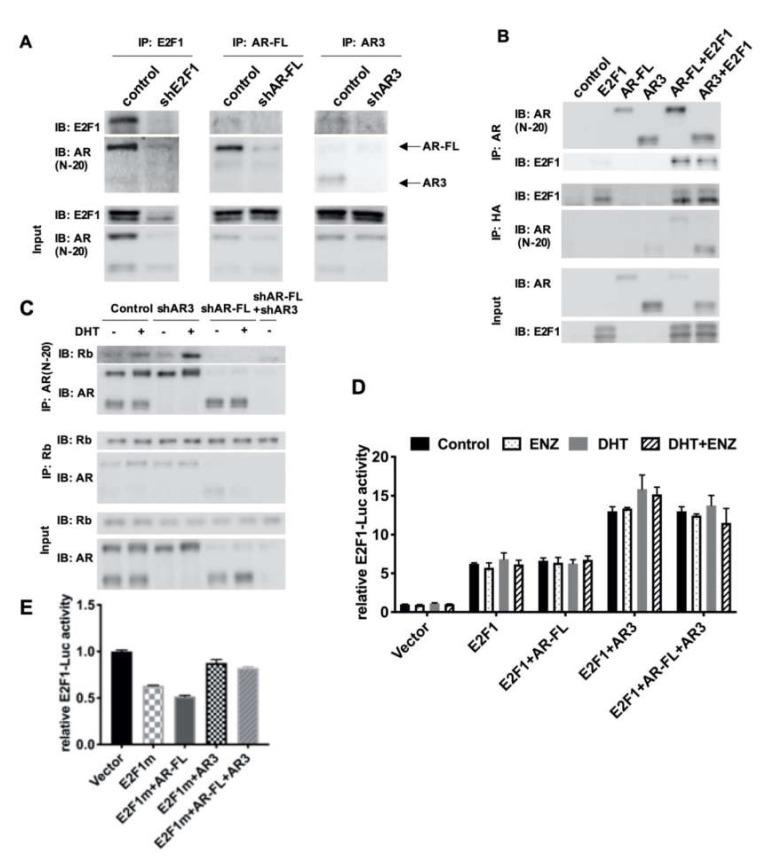
AR3 and AR-FL recruited different cofactors and differentially regulated E2F1 gene expression. (**A**) R1-ADR cells were infected with the lentivirus encoding the control shRNA, shAR3 or shAR-FL for 48 h. Cell lysates were then immunoprecipitated with an anti-AR or anti-E2F1 antibody followed by western blot. (**B**) HEK293T cells were cotransfected with AR-FL, AR3, and/or E2F1-HA. Cell lysates were then immunoprecipitated with an anti-AR or anti-HA antibody followed by western blot. (**C**) R1-ADR cells were infected with the lentivirus encoding the control shRNA or shAR3 or shAR-FL for 48 h and then treated with 10nM DHT for 2 h before collection. Cell lysates were then immunoprecipitated with an anti-AR or anti-Rb antibody followed by western blot. (**D**) E2F1-promoter luciferase assay was performed in HEK293T cells treated with 0.1nM DHT or/and 10 μM ENZ. (**E**) E2F1-promoter luciferase assays with mutant E2F1 were performed in HEK293T cells.

Next, we aimed to investigate how AR-FL and AR3 differentially regulate E2F1 in R1-ADR cells. It has been reported that E2F1 interacts with AR-FL directly, in either an androgen-dependent or androgen-independent manner [13,29]. Interestingly, both E2F1 activation signaling and AR activation signaling are positively correlated and overlap both in CRPC cell lines and patient samples [10,30]. Moreover, a recent report showed that AR can repress the expression of a subset of E2F-regulated genes by recruiting hypo-phosphorylated Rb upon DHT stimulation [16]. Thus, it is possible that AR3 and E2F1 cooperatively regulate gene expression, including the regulation of E2F1 itself via the differential recruitment of transcriptional co-factors in comparison to AR-FL. To test this hypothesis, first, we performed co-immunoprecipitation assays to determine whether E2F1 was associated with AR-FL or AR3 in R1-ADR cells. Figure 4A shows that both endogenous AR-FL and AR3 were able to be co-immunoprecipitated with, and therefore interact with, E2F1 in R1-ADR cells. Such interactions were also confirmed when E2F1 was overexpressed with AR-FL or AR3 in HEK293T cells (Figure 4B), suggesting a direct interaction between E2F1 and AR-FL or AR3.

Previous studies have shown that AR-FL interacted with Rb, and that binding was enhanced by DHT treatment in exogenous overexpression HEK293T cells [16,31]. Next, we examined the interaction between Rb with AR-FL or AR3 in R1-ADR cells. In agreement with previous reports, AR-FL interacted with Rb and this interaction was enhanced by DHT (Figure 4C). However, AR3 was not co-immunoprecipitated with Rb, suggesting that Rb selectively binds to AR-FL in these cells. Furthermore, it has been shown that E2F1 can be positively self-regulated by binding to its own promoter [20]. Thus, we examined whether AR3 and E2F1 regulate E2F1 promoter using the E2F1-LUC reporter. Indeed, AR3+E2F1 enhanced luciferase activity compared to E2F1 alone (Figure 4D, Figure S4A). Conversely, AR-FL had little effects on the reporter. To test whether the effects of AR3 on the E2F promoter depends on E2F1, we co-expressed the mutant E2F1 plasmid with no DNA binding activity with AR-FL or AR3 and examined their activity on the E2F1-LUC reporter. We found that in the presence of the DNA binding mutant E2F, AR3 failed to enhance the reporter (Figure 4E, Figure S4B). Taken together, these findings suggest that AR3 and AR-FL recruit different cofactors and only the AR3+E2F1 complex regulates E2F1 expression.

### 3.5. E2F1 Regulates AR Gene Expression and Forms a Positive Feedforward Loop with AR3

**Figure 5:**
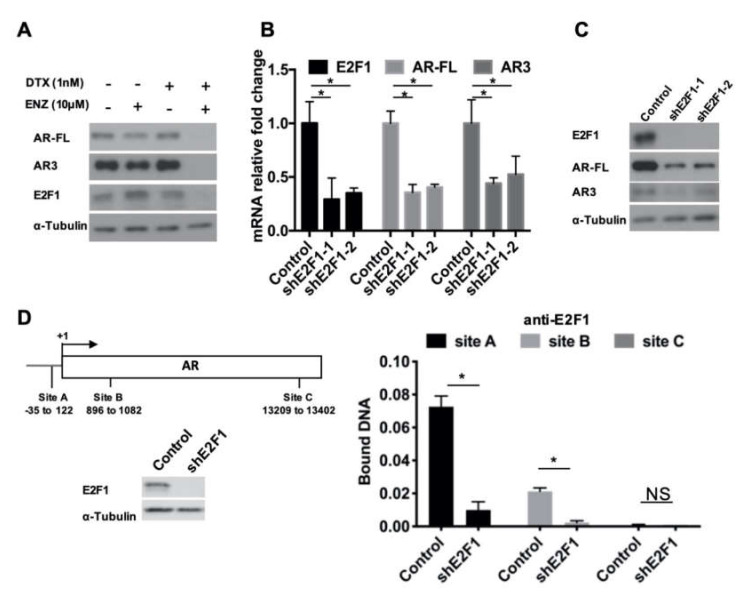
E2F1 regulated AR expression. (**A**) AR-FL, AR3, and E2F1 protein level 4 days after DTX+ENZ treatment were determined by western blot. (**B**,**C**) R1-ADR cells were infected with the lentivirus encoding the control shRNA or E2F1 shRNA for 48 h. Expression level of E2F1, AR-FL, and AR3 were determined by qRT-PCR (**B**), Error bars, S.D. * *p* < 0.05 and western blot (**C**). (**D**) Binding of E2F1 to the regulatory regions of AR was analyzed by ChIP assay.

Because ENZ is known to target AR-FL, we also examined AR-FL and AR3 expression after treatment with DTX+ENZ for four days. Figure 5A shows that the protein level of AR-FL and AR3 decreased along with that of E2F1. As it has been previously shown that E2F1 regulated AR expression [32], it is possible that the combined treatment induced the downregulation of E2F1, which could in turn have caused a reduction in both AR-FL and AR3. To test this hypothesis, we knocked down E2F1 using two E2F1-specific shRNAs. The expression of AR-FL and AR3 were examined by qRT-PCR and western blot. The results show that both AR-FL and AR3 mRNA and protein expression decreased upon E2F1 knock-down in R1-ADR cells (Figure 5B, C). We also examined the effect of E2F1 knock-down in other cell lines. Similarly, we found that both AR-FL and AR3 expression were decreased by E2F1 knock-down (Figure S5A). Controversially, Davis et al. [33] demonstrated that the overexpression of E2F1 inhibited AR mRNA and protein expression in LNCaP cells. A more recent study from the same group showed that E2F1 and DNMT1 co-occupied the AR promoter and repressed AR expression [32]. To resolve the apparent discrepancy, we over-expressed E2F1 at various concentrations in R1-ADR cells and examined their effects on AR gene expression. In agreement with previous studies, E2F1 inhibited both AR-FL and AR3 expression at higher levels of E2F1 (Figure S5B). However, at relatively lower levels, E2F1 was able to promote AR-FL and AR3 expression. This finding suggests that the regulation of AR gene expression by E2F1 is dependent on E2F1 abundance, and is possibly dictated by the availability of its co-factor(s).

We further examined whether E2F1 could be recruited to the AR promoter. We performed ChIP assays using a primer set for the E2F1 binding sites (sites A, B, and C) on AR promoter described by Valdez et al. [32]. In comparison to the E2F1 knock-down group, the control exhibited much higher E2F1 ChIP binding affinity to sites A and B (Figure 5D). Thus, these data indicate that E2F1 directly regulates the expression of AR in drug-resistant prostate cancer cells, and such regulation is possibly context and concentration dependent.

### 3.6. AR and E2F1 Expression were Recovered in DTX-ENZ Resistant Prostate Cancer Cells

**Figure 6:**
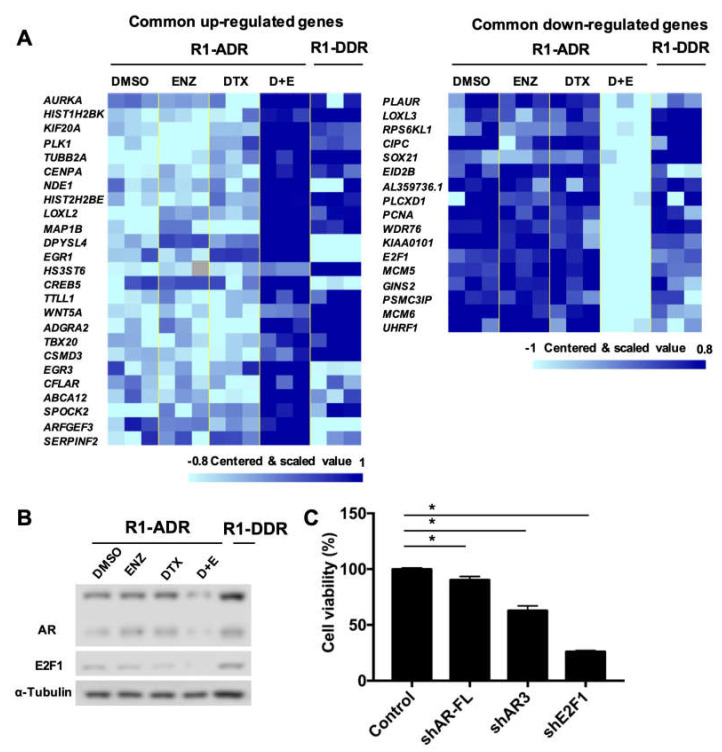
E2F1 and AR/ARv expression was recovered in R1-DDR cells. (**A**) RNA-seq analysis, heatmap of those commonly dysregulated genes in R1-ADR cells treated with DTX+ENZ (p < 0.05, FDR < 0.25, fold change >1.4), we compared R1-DDR cells (maintained in DTX+ENZ) to R1-ADR cells treated with DTX+ENZ (D+E). Missing values are in color “gray”. (**B**) protein level of AR/ARv and E2F1 in R1-DDR cells were determined by western blot. (**C**) R1-DDR cells infected with lentivirus encoding control or shE2F1 or shAR-FL or shAR3. Cell growth was determined by CCK-8 assays 3 days after infection.

Cross-resistance is common in CRPC patients [34,35]. We found cells treated with DTX+ENZ eventually acquired resistance to both drugs after approximately three months long-term culture. Thus, we established DTX-ENZ double drug-resistant prostate cancer cell lines, R1-DDR and LN-DDR, by long term culturing R1-ADR and LN-ADR in medium containing both drugs, respectively. We performed an RNA-seq analysis and compared R1-DDR cells to R1-ADR cells treated with DTX+ENZ. Figure 6A shows that the expression of most of the commonly dysregulated genes in R1-ADR cells treated with DTX+ENZ, including E2F1, were restored in the double drug-resistant R1-DDR cells (Figure 6A). We further validated the protein expression of AR/ARv and E2F1 by western blot and found that the protein levels of both AR/ARv and E2F1 were recovered in R1-DDR cells (Figure 6B). To assess whether E2F1 and AR are critical in double drug-resistant cell growth, we knocked down AR-FL, AR3, or E2F1 by target-specific shRNA in R1-DDR. Cell growth was consequently inhibited by knocking down the target genes (Figure 6C). Together, these data suggest that the recovery of E2F1 and AR signaling plays an important role in the survival of cells in the DTX-ENZ double drug-resistant cell lines.

### 3.7. Auranofin Suppressed E2F1 and AR3 Expression and Inhibited Double Drug-Resistant Cell Growth In Vitro and In Vivo

**Figure 7:**
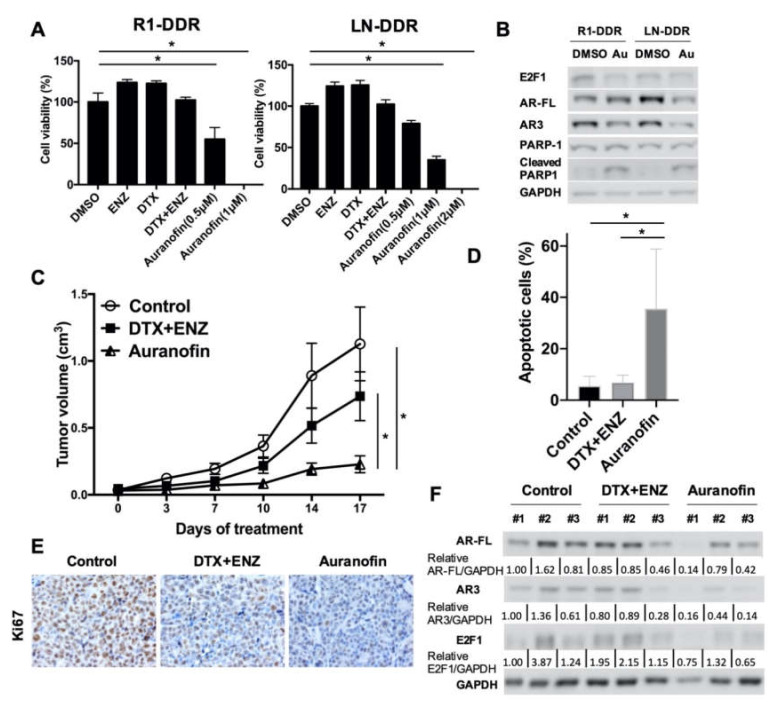
Auranofin inhibited the growth of double drug-resistant prostate cancer cells in vitro and in vivo. (**A**) R1-DDR (left panel) and LN-DDR (right panel) cells were treated with different doses of Auranofin for 72 h. Cell growth was determined by CCK8 assay. (**B**) R1-DDR and LN-DDR cells were treated with 2 μM Auranofin for 12 h, Protein level of E2F1, AR-FL, and AR3 in R1-DDR cells were determined by western blot. (**C**) Animals were treated with solvent or DTX (5 mg/kg once a week) + ENZ (10 mg/kg per day, 5 days per week) or auranofin (5 mg/kg per day, 5 days per week), n = 5 per group. Error bars, S.E.M. * *p* < 0.05 (**D**) TUNEL assay was performed to detect apoptosis of R1-DDR xenograft. Error bars, S.D. * *p* < 0.05. (**E**) Ki67 proliferation marker was examined by immunohistochemistry. (**F**) AR/ARv and E2F1 protein level in R1-DDR xenograft were determined by western blot.

In a search for potential therapeutic agents to overcome double drug-resistance, we found that auranofin, a gold-based compound that is an FDA-approved drug for the treatment of rheumatoid arthritis, efficiently inhibited double drug-resistant cell growth (Figure 7A). In recent years, the repurposing of auranofin has been investigated for a number of diseases, including neurodegenerative disorders, HIV, infections, and cancer [36]. A recent study showed that auranofin promoted AR protein degradation and inhibited AR transcription, which in turn suppressed AR-positive prostate cancer cell survival [37]. Given the fact that AR-FL played a limited role in our drug resistant cells, we speculate that there could be additional mechanisms of auranofin-induced growth inhibition. To further evaluate the effect of auranofin on monotherapy-resistant cells, we measured the growth inhibition of DTX-resistant R1-ADR cells and ENZ-resistant R1-ENZR cells. The results showed that auranofin inhibited growth in both cell lines in a similar manner (Figure S6A). As we have already shown that DTX+ENZ inhibited drug-resistant cell growth through downregulation of the AR3-E2F1 feedforward loop, we examined the expression of AR3 and E2F1 in response to auranofin treatment in double drug-resistant cells by western blot and qRT-PCR. The results show that the levels of AR3 and E2F1 protein and mRNA were inhibited by auranofin in both R1-DDR and LN-DDR cells (Figure 7B, Figure S6B). These data suggest that auranofin is able to decrease the AR3-E2F1 axis and inhibit the growth of resistant cells.

We also examined the effects of auranofin on double drug-resistant cells *in vivo*. As shown in Figure 7C and Figure S6C, the volume and size of R1-DDR tumors in mice were significantly decreased as a result of auranofin treatment in comparison to either the vehicle control or the DTX+ENZ treatment group. Relative body weight did not change significantly within the groups (Figure S6D). TUNEL assays showed that auranofin-treated xenograft tumors exhibited higher numbers of apoptotic-positive cells (Figure 7D, Figure S6E). Immunohistochemistry staining showed that the proliferation marker, Ki67, was lower in response to treatment with auranofin (Figure 7E). Western blotting showed that protein levels of E2F1 and AR/ARv tend to be lower in auranofin-treated tumors in comparison to controls or the DTX+ENZ group (Figure 7F). Collectively, these findings suggest that auranofin decreased E2F1 and AR3 expression and is a potent inhibitor of cell growth in double drug-resistant prostate cancer cells both in vitro and in vivo.

## 4. Discussion

To elucidate the molecular mechanisms and investigate new strategies for delaying and circumventing drug resistance, we developed cell models that were resistant to either DTX or ENZ. We tried several combinations of a variety of drugs and found that only DTX+ENZ effectively reduced the survival of the drug-resistant cells. This finding is consistent with a previous report which showed antiandrogens inhibited drug efflux activity and reversed docetaxel resistance in advanced prostate cancer [38]. In order to further investigate the underlying mechanisms responsible for the efficacy of the combined treatment, we performed RNA-Seq to detect the early-stage molecular events that were elicited in response to this combination. We found that E2F1 is a key regulator and was downregulated by the combined treatment. E2F1 is a known dual-function cancer progression mediator. It has been shown that while an excess amount of E2F1 triggers apoptosis, under certain signaling conditions, E2F1 can shift gears to pro-survival [11]. The increased expression of E2F1 has been known to occur in many types of cancers, including prostate cancer. We analyzed E2F1 profiling using two public databases (Trento/Cornell/Broad 2016 and the TCGA prostate adenocarcinoma, provisional), and found that E2F1 is either amplified or elevated in a significant portion of patients [39,40,41].

AR signaling, especially the AR variant AR3/AR-V7-driven molecular events, are critical for therapeutic resistance in aggressive prostate cancer. While the expression and activities of E2F1 and AR are often correlated, the details of this co-regulation are still not fully understood. It has been reported that a low dose of androgen induced E2F activity, while a high dose of androgen inhibited E2F activity [42]. One explanation of this bidirectional regulation is that the high dose of androgen recruited Rb to common AR and E2F binding sites on the chromatin, and thus repressed the expression of genes regulated by the AR-E2F axis (including E2F1 itself) [16]. In the present study, we demonstrated that AR3 did not co-immunoprecipitate with Rb but AR-FL did. AR3 interacted with E2F1 and bound to the promoter region of E2F1. It is also worth noting that the AREs that we identified in the E2F1 regulatory region are half AREs, and therefore may only allow the AR variant to bind in the presence of E2F1. This possibility is supported by our luciferase reporter assays, which show that AR3, but not AR-FL, can increase E2F1-luciferase activity (Figure 3D). The addition of AR-FL to AR3 exerted no effect on E2F1-luciferase activity. These data could indicate that the activity of AR3 on E2F1 is not dependent on AR-FL. As expected, we also found that AR3 alone was able to induce ARR2-luciferase activity without the presence of AR-FL. This induction is not affected by treatment with either DHT or ENZ. However, ENZ does inhibit AR3 activity when AR-FL is co-expressed, and this inhibition is increased concomitantly with AR-FL expression. This finding is consistent with a previous study, which reported that the activity of constitutively active AR variants required AR-FL [28]. A very recent study showed that AR3 and AR-FL bind to the same sites on chromatin and regulate H3K27ac [43]. Furthermore, a GSEA indicated that E2F1 targets are co-regulated by both AR3 and AR-FL. On the other hand, He et al. [44] reported distinct epigenome landscapes at ARv preferential binding sites and AR-FL preferential binding sites in CRPC cells. Another recent study showed that AR3, but not AR-FL, colocalized with HoxB13 on open chromatin and regulated target gene expression [45]. The ChIP-seq results from another study showed that a significant portion of AR3 binding sites lacked AR-FL binding, BRD4 and ZFX co-occupies with AR3 on the AR3-unique binding sites of target gene promoters [46]. Thus, it will be interesting to study whether the epigenetic modification or co-binding factors determine the distinct role of AR3 in different genetic contexts in the future. It should be noted that our study was mainly carried out in preclinical models. It has yet to be validated whether such mechanisms are applicable for predicting patients with acquired resistance.

Several recent reports have shown that ENZ is able to radiosensitize prostate cancer in vitro and in vivo by impairing the DNA damage response [47,48]. Similarly, it is possible that there is a high level of DNA damage response in DTX and ENZ monotherapy-resistant cells. The combined treatment simultaneously diminished AR signaling and E2F1 expression. E2F1—a dual role regulator—is important in cell cycle progression, the DNA damage response, and apoptosis, and either the upregulation or loss of E2F1 can promote DNA damage-induced apoptosis [49,50]. Interestingly, the AR signaling pathway regulates a set of DNA repair genes, while anti-androgenic treatments cause increases in DNA damage [51]. It has also been shown that AR variants interacted with DNA-PKs in response to DNA damage [52]. Blocking the interaction of AR variants and DNA-PKs causes persistent DNA damage and cell death during radiation therapy. A recent report demonstrated that the loss of AR-mediated CDC6 expression alteration resulted in an accumulation of DNA damage and promoted apoptosis [53]. More importantly, E2F1 and AR signaling have many common target genes, including genes associated with DNA damage repair and DNA replication [16,30,51]. Thus, it is very likely that the loss of both E2F1 and AR3 causes DNA damage and an increase in apoptosis in the combined treatment. We will examine whether the AR3-E2F1 axis plays a role in the DNA damage response in a future study.

In this study, we generated DTX and ENZ double drug-resistant prostate cancer cells, which were highly resistant to several compounds that we tested. However, we found that auranofin—an FDA-approved drug for the treatment of rheumatoid arthritis—effectively inhibited double drug-resistant prostate cancer cell growth in vitro and in vivo. Furthermore, this effect is at least partially a result of the downregulation of the E2F1-AR3 axis. The main mechanism of action of auranofin is via the inhibition of redox enzymes. Increasing evidence shows that auranofin has anti-cancer effects [36]. A recent study showed that auranofin inhibited proteasomal deubiquitinases and facilitated the degradation of AR [37]. In this study, we found that both the mRNA and protein levels of AR/ARv and E2F1 were reduced in response to treatment with auranofin, indicating an additional mechanism of action for auranofin. It has been shown that the auranofin target TrxR/Trx binds directly to PTEN and inhibits its lipid phosphatase activity [54]. Another report showed that auranofin regulates the phosphorylation status of AKT and H2AX [55]. Interestingly, we found the phosphorylation of both AKT and H2AX was altered in both DTX+ENZ and auranofin treatments (data not shown). Thus, it will be interesting to further investigate how these treatments affect the E2F1-AR3 axis in the future.

## 5. Conclusions

In summary, this is a proof-of-principle study to demonstrate that simultaneously targeting E2F1 and AR3 via a combined therapy or a multi-targeting drug could circumvent castration resistance in prostate cancer. We also showed that the differential regulation of E2F1 expression by AR3, but not by AR-FL, in R1-ADR cells, suggesting that AR3 may function independently of AR-FL in different genetic contexts via the differential recruitment of co-factors.

## Supplementary Materials

The following are available online at https://www.mdpi.com/2073-4409/9/5/1094/s1, The results of Figure S4 are in whole or part based upon data generated by the TCGA Research Network: http://cancergenome.nih.gov/.
