# Supplementary material for: The Role of Crosstalk between AR3 and E2F1 in Drug Resistance in Prostate Cancer Cells
Source: Cells. 2020 Apr 28;9(5):1094. doi: 10.3390/cells9051094 (PMC7290672; doi:10.3390/cells9051094)
Supplement: Supplementary file 1 [file cells-09-01094-s001.pdf]

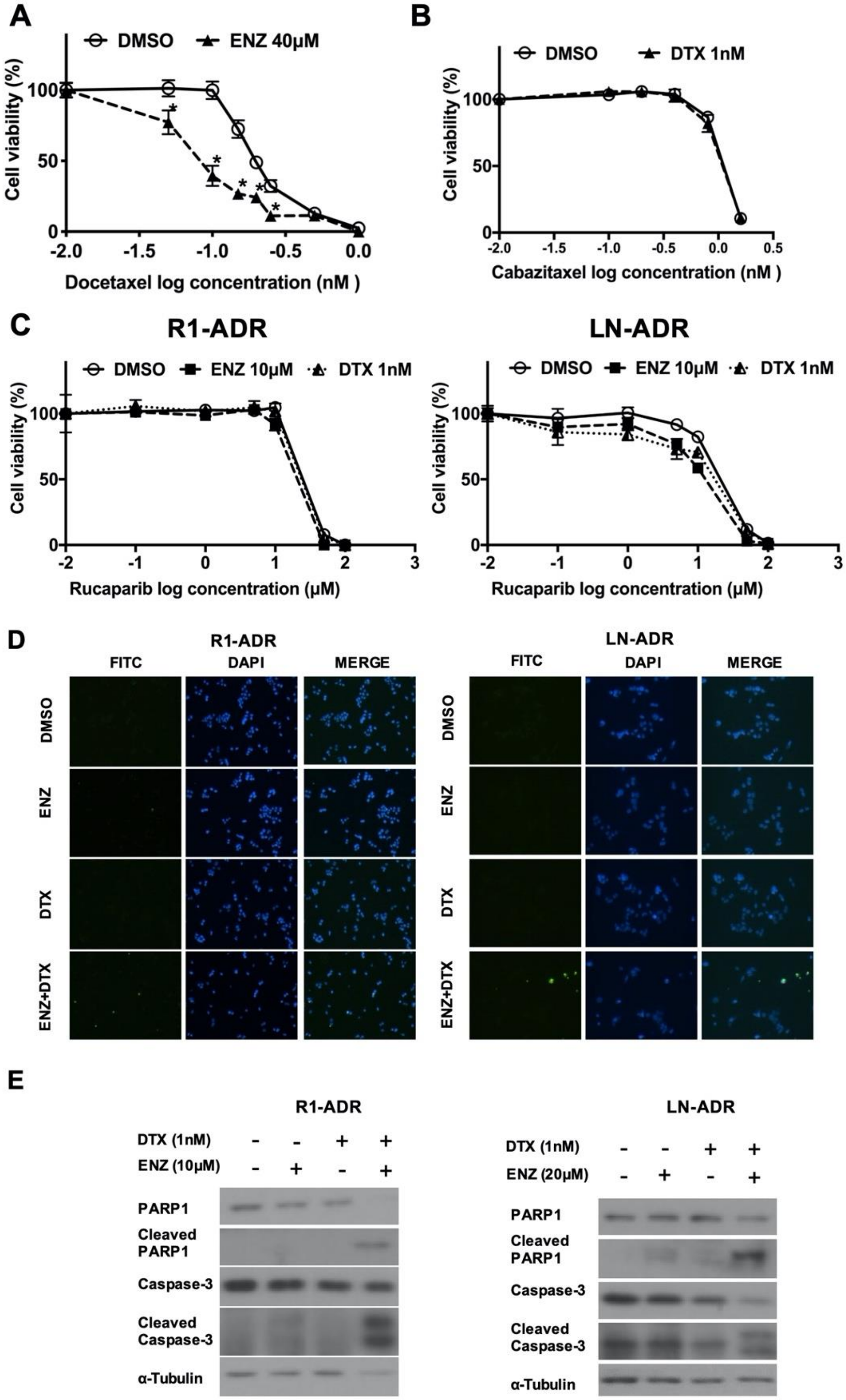

Supplementary Figure S1. Combined DTX and ENZ inhibited growth and induced apoptosis of drug resistant cells. A, The presence of ENZ enhanced the growth inhibition effect of DTX in ENZ-resistant R1-ENZR cells. R1-ENZR cells were treated with different doses of DTX for 3 days with the presence 40µM ENZ. Error bars, S.D. *, p<0.05. B, Combined DTX + cabazitaxel has

little or no enhanced effect compared to cabazitaxel alone. R1-ADR cells were treated with different doses of cabazitaxel for 4 days with the presence 1nM DTX. C. ENZ or DTX has little or no enhanced effect in combination with PARP inhibitor Rucaparib compared to Rucaparib alone. R1-ADR (left panel) and LN-ADR (right panel) cells were treated with different doses of Rucaparib for 4 days with the presence of 10μM ENZ or 1nM DTX. D, DAPI and TUNEL-FITC staining of R1-ADR (left panel) and LN-ADR (right panel) cells treated with DTX and/or ENZ for 48 hours. E, Protein level of cleaved PARP1 and cleaved caspase-3 in R1-ADR (left panel) and LN-ADR (right panel) cells treated with DTX and/or ENZ for 48 hours. Cells were treated with 1nM DTX or/and 10μM ENZ (R1-ADR) or 20μM ENZ (LN-ADR).

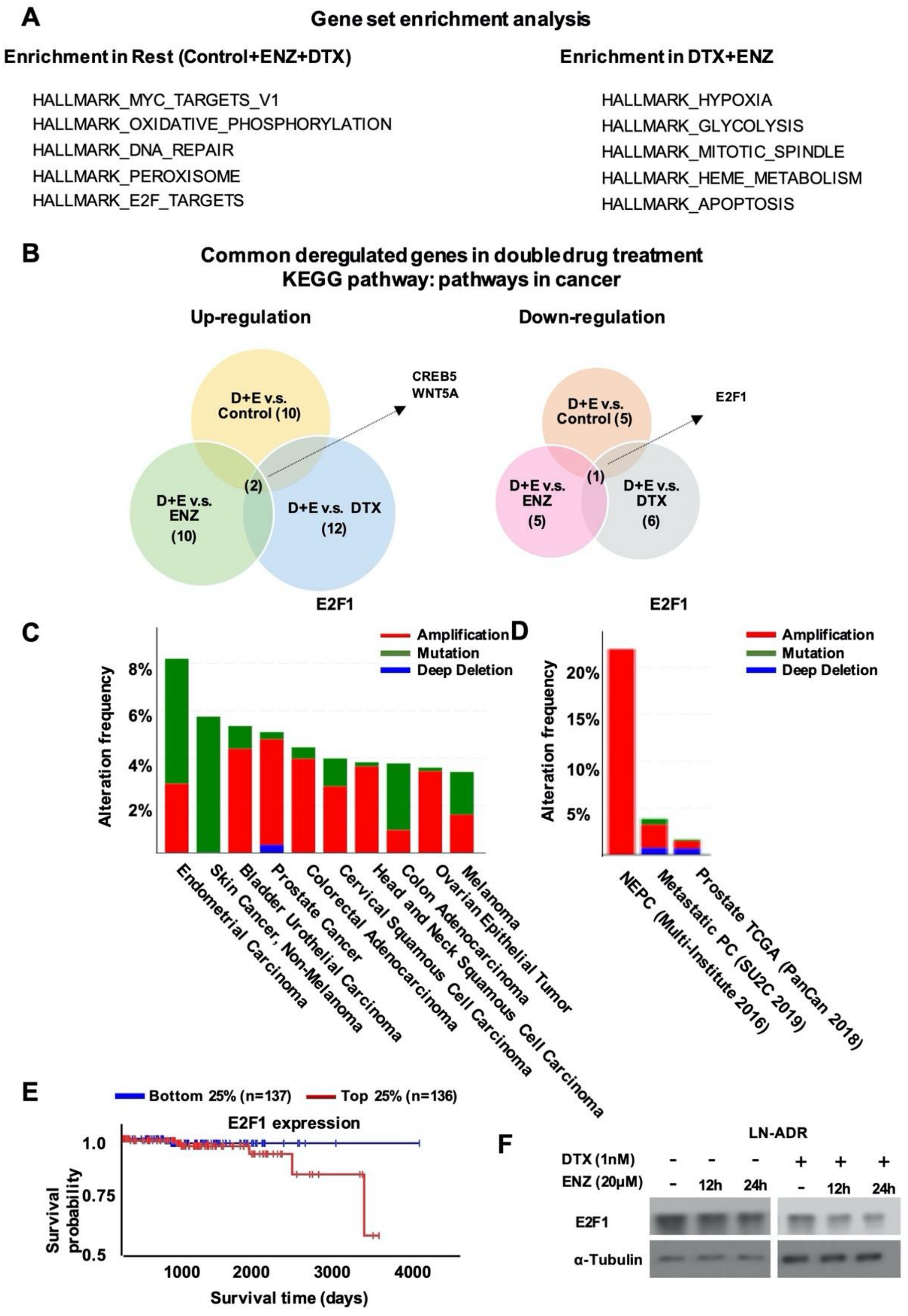
A
Gene set enrichment analysis
Enrichment in Rest (Control+ENZ+DTX)
HALLMARK_MYC_TARGETS_V1
HALLMARK_OXIDATIVE_PHOSPHORYLATION
HALLMARK_DNA_REPAIR
HALLMARK_PEROXISOME
HALLMARK_E2F_TARGETS
Enrichment in DTX+ENZ
HALLMARK_HYPOXIA
HALLMARK_GLYCOLYSIS
HALLMARK_MITOTIC_SPINDLE
HALLMARK_HEME_METABOLISM
HALLMARK_APOPTOSIS
B
Common deregulated genes in doubledrug treatment
KEGG pathway: pathways in cancer
Up-regulation
D+E v.s. Control (10)
D+E v.s. ENZ (10)
D+E v.s. DTX (12)
(2)
CREB5
WNT5A
Down-regulation
D+E v.s. Control (5)
D+E v.s. ENZ (5)
D+E v.s. DTX (6)
(1)
E2F1
C
E2F1
Amplification
Mutation
Deep Deletion
Alteration frequency
8%
6%
4%
2%
Endometrial Carcinoma
Skin Cancer, Non-Melanoma
Bladder Urothelial Carcinoma
Prostate Cancer
Colorectal Adenocarcinoma
Cervical Squamous Cell Carcinoma
Head and Neck Squamous Cell Carcinoma
Colon Adenocarcinoma
Ovarian Epithelial Tumor
Melanoma
D
E2F1
Amplification
Mutation
Deep Deletion
Alteration frequency
20%
15%
10%
5%
NEPC (Multi-Institute 2016)
Metastatic PC (SU2C 2019)
Prostate TCGA (PanCan 2018)
E
Bottom 25% (n=137)
Top 25% (n=136)
E2F1 expression
Survival probability
1.0
0.75
0.5
1000
2000
3000
4000
Survival time (days)
F
LN-ADR
DTX (1nM) - - - + + +
ENZ (20μM) - 12h 24h - 12h 24h
E2F1
α-Tubulin

Supplementary Figure S2. Differential gene expression in response to DTX+ENZ combined treatment. A, Gene set enrichment analysis of DTX+ENZ v.s. Rest (Control+ENZ+DTX). Top 5 gene sets with lowest FWER p-value were presented. B, Analysis of common deregulated genes in DTX+ENZ v.s. Control or ENZ or DTX group in KEGG pathways in cancer gene list . (p<0.05, FDR<0.25, fold change > 1.4) C, Cancer types summary, E2F1 alteration frequency in various cancer types (Curated set of non-redundant studies, minimum 100 total cases per cancer type, minimum 3% altered cases). Data was acquired and analyzed from cBioPortal. D, E2F1 alteration frequency in selected prostate cancer studies. Data was acquired and analyzed from cBioPortal. E, Kaplan-Meier overall survival analysis of TCGA prostate adenocarcinoma database. P-value = 0.2800, Log-rank test statistics = 1.167. Data was acquired and analyzed from UCSC xena. F, Protein expression of E2F1 in LN-ADR cells 12 and 24 hours after drug treatment were determined by western blot.

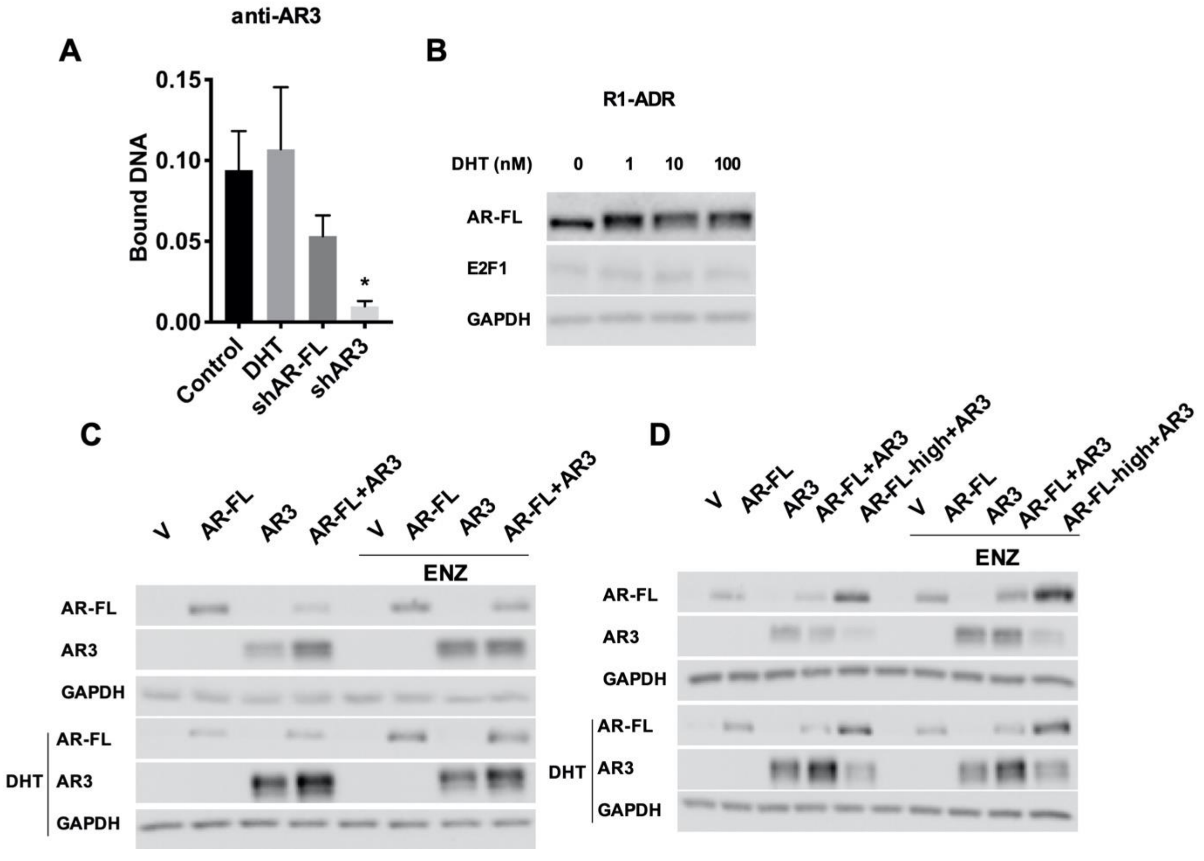

Supplementary Figure S3. AR3 and AR-FL differentially regulated E2F1 expression A, Binding of AR3 to the known ARE site of human FKBP5 gene was analyzed by ChIP assay and used as a positive control. B, R1-ADR cells were treated with various doses of DHT for 24 hours. AR-FL and E2F1 protein level were determined by western blot. C-D, Protein expression of AR-FL, and AR3

in figure 3. D (C in this supplementary), E (D in this supplementary) were confirmed by western blot with GAPDH as a loading control.

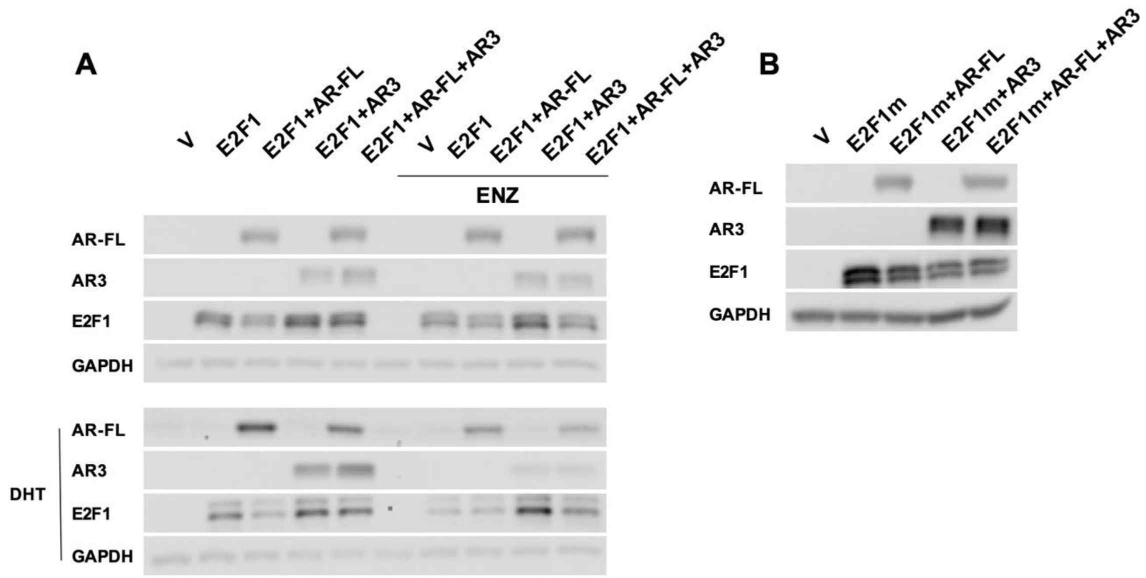

Supplementary Figure S4. Expression of AR-FL, AR3, and E2F1 in figure 4. D (A in this supplementary), E (B in this supplementary) were confirmed by western blot with GAPDH as a loading control.

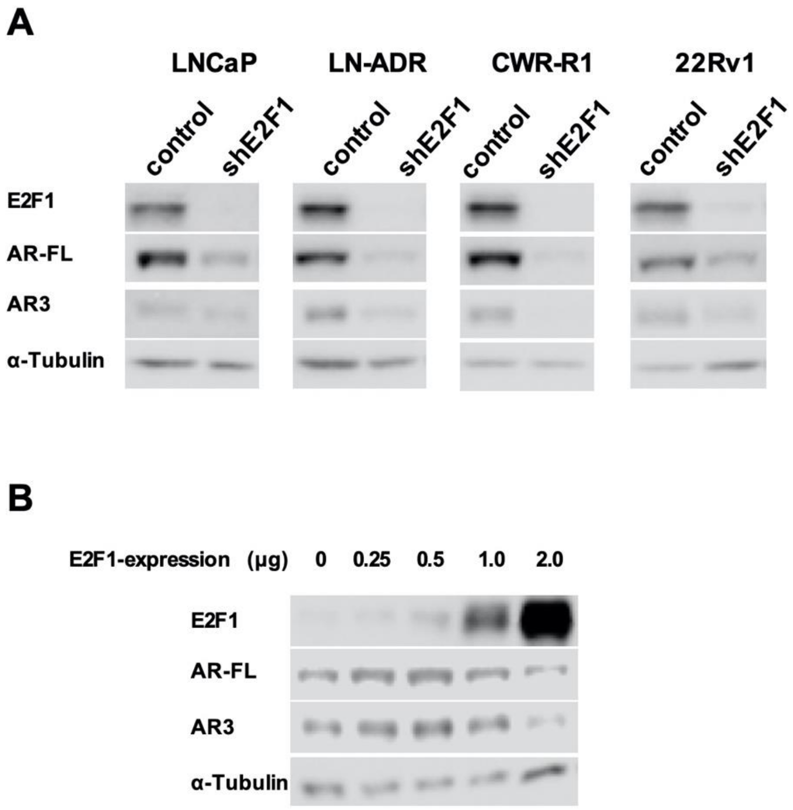

Supplementary Figure S5. E2F1 regulated AR-FL and AR3 expression. A, Cells were infected with the lentivirus encoding the control shRNA or E2F1 shRNA for 48 hours. Protein level of E2F1, AR-FL, and AR3 were determined by western blot. B, Different doses of E2F1 plasmids were transfected into R1-ADR cells and cultured for 48 hours. Expression level of E2F1, AR-FL, and AR3 were determined by western blot.

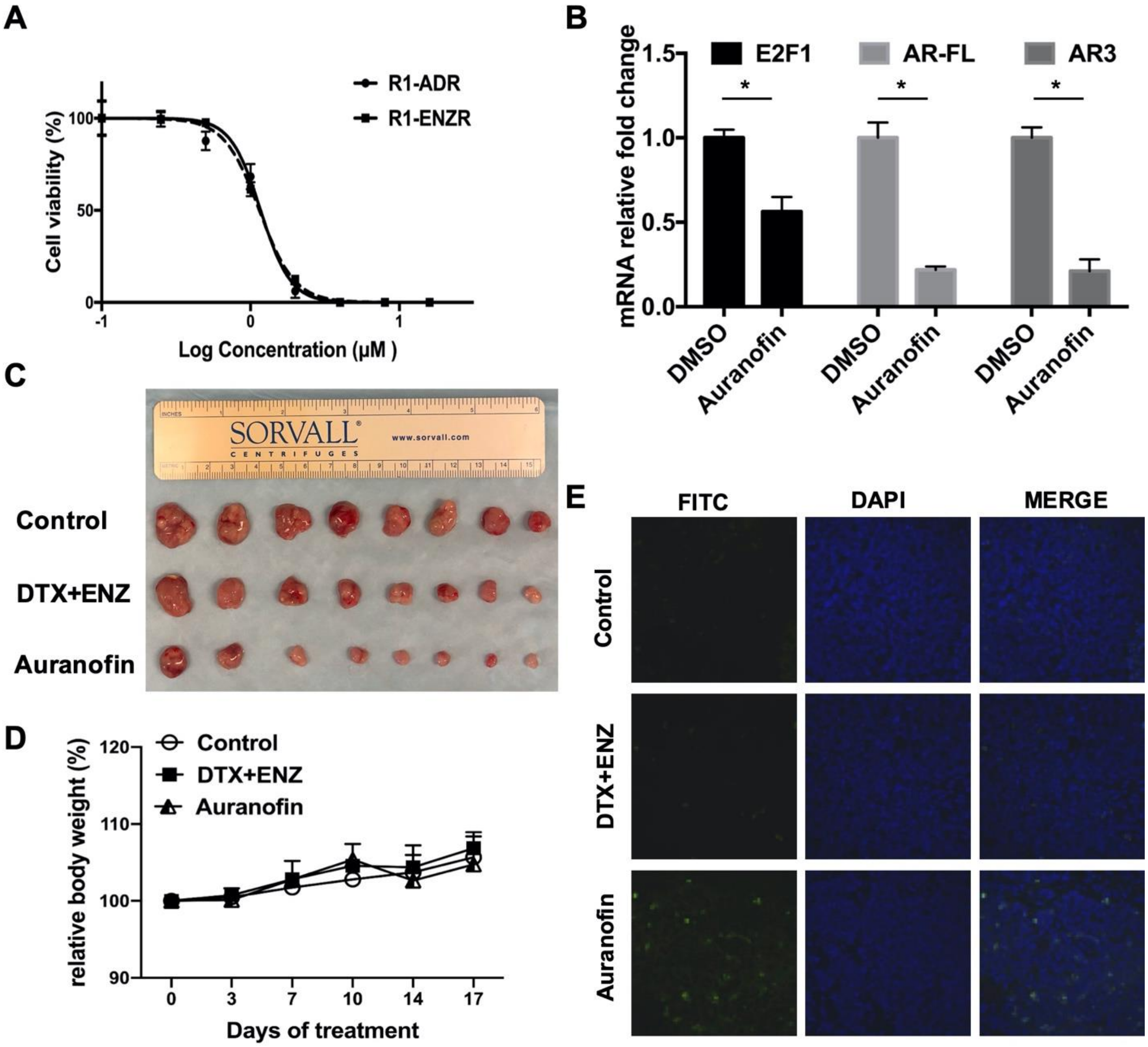

Supplementary Figure S6. Auranofin suppressed double drug-resistant cell growth and reduced AR and E2F1 expression. A, R1-ADR and R1-ENZR cells were treated with different doses of Auranofin for 72 hours. Cell growth was determined by CCK8 assay. B, R1-DDR cells were treated with 2μM Auranofin for 12 hours, RNA level of E2F1, AR-FL, and AR3 in R1-DDR cells were determined by qRT-PCR, Error bars, S.D. *, $p<0.05$ C, Photograph of R1-DDR xenograft tumors. D, relative body weight analysis by repeated measures ANOVA with group as between subject factor and time as within subject factor and no significant group difference was detected ($p = 0.889$). E, DAPI and TUNEL-FITC staining of R1-DDR xenograft tumors.
